# Do regional characteristics predict developmental delay? Analyses of German school entry examination

**DOI:** 10.1093/eurpub/ckac129.342

**Published:** 2022-10-25

**Authors:** S Hoffmann, M Tschorn, N Michalski, J Hoebel, BR Förstner, MA Rapp, J Spallek

**Affiliations:** 1 Department of Public Health, Brandenburg University of Technology CB-SFB, Senftenberg, Germany; 2 Faculty of Health Sciences Brandenburg, Research Area Services Research and e-Health, Potsdam, Germany; 3 Department of Sports and Health Sciences, Faculty of Human Science, University Potsdam, Potsdam, Germany; 4 Division of Social Determinants of Health, Robert Koch Institute, Berlin, Germany

## Abstract

**Background:**

Children's health and development are strongly linked to their living situation, including their family's socioeconomic position (SEP) and living region. However, research on the impact of the living region on children's development beyond family SEP is scarce. This study evaluated whether rurality and regional socioeconomic deprivation (DEP) are associated with children's development independently of family SEP.

**Methods:**

The study used population-based data of 5-6.5 years old children (n = 22,801) from mandatory school entry examinations (SEE) in the German federal state of Brandenburg, which were examined in 2018/2019. The SEE data have been linked with data on i. rurality that was defined by an inverted population density and ii. regional DEP that were provided by the German Index of Socioeconomic Deprivation. By binary multilevel models, the predictive values of rurality and regional DEP for global developmental delay (GDD) were evaluated, while adjusting for family SEP.

**Results:**

Children with high family SEP showed reduced odds for GDD compared to medium family SEP (female: OR = 4.26, CI95=3.14-5.79, male: OR = 3.46, CI95=2.83-4.22) and low family SEP (female: OR = 16.58, CI95=11.90-23.09, male: OR = 12.79, CI95=10.13-16.16). Regional DEP additionally predicted GDD, with higher odds for children from more deprived regions (female: OR = 1.35, CI95=1.13-1.62, male: OR = 1.20, CI95=1.05-1.39). Rurality did not predict GDD beyond family SEP and regional DEP.

**Conclusions:**

In addition to family SEP, the regional DEP has an effect on children's developmental delay. Hence, Public Health should take into account regional socioeconomic conditions as determinant of health over the life course in addition to family SEP.

**Key messages:**

• Regional socioeconomic deprivation contributes to inequalities in children's development and health.

• Besides family SEP, regional socioeconomic circumstances are of particular interest to promote health over the life course.

